# Supplementary material for: Maternal high-fat diet in mice induces cerebrovascular, microglial and long-term behavioural alterations in offspring
Source: Commun Biol. 2022 Jan 11;5:26. doi: 10.1038/s42003-021-02947-9 (PMC8752761; doi:10.1038/s42003-021-02947-9)
Supplement: Supplementary file 2 — Supplementary Information [file 42003_2021_2947_MOESM2_ESM.pdf]

Supplementary

Supplementary methods

Ultrastructure (supplementary detailed protocol)

Immuno-electron microscopy tissue processing

Sections were washed in phosphate-buffered saline (PBS), quenched in 0.3% H_2O_2 (in PBS) for 10 minutes, washed, permeabilized in 0.1% $NaBH_4$ (in PBS) for 30 minutes, and washed again. After quenching and permeabilization, sections were placed for 1 hour at room temperature (RT) in blocking buffer (10% fetal bovine serum, 3% bovine serum albumin, 0.01% Triton X-100 in 50mM TBS, pH=7.6) and incubated overnight at 4°C with the primary antibody rabbit anti-Iba1 (1:1000 in BB; cat#019-19741, FUJIFILM Wako Chemical, Osaka, Japan). The next day, brains sections were washed in Tris-buffered saline (TBS), incubated for 1.5 hours at RT with the secondary antibody biotinylated goat anti-rabbit (1:300 in TBS; cat# 111-066-046, Jackson ImmunoResearch, West Grove, PA, United States), followed by the avidin-biotin complex (1:1:100 in TBS; cat# PK-6100 ,Vector Laboratories, Burlingame, CA, United States) for 1 hour at RT, then washed and revealed in 0.05% diaminobenzidine (DAB, 0.015% H_2O_2, in TBS; cat# D5905-50TAB, MilliporeSigma). Sections were next processed for electron microscopy (EM). Tissues were incubated in 3% ferrocyanide (in H_2O; cat# PFC232.250, BioShop, Burlington, ON, Canada) combined (1:1) with 4% aqueous osmium tetroxide (cat#19170, Electron Microscopy Sciences, Hatfield, PA, United States) for 1 hour, washed in PBS, incubated in 1% thiocarbohydrazide (in PBS; cat# 2231-57-4, Electron Microscopy Sciences) for 20 minutes, washed in PBS, incubated in 2% osmium tetroxide (in H_2O), then dehydrated in ascending concentration of ethanol (35%, 50%, 70%, 80%, in 90%, in 100%) followed by incubation in propylene oxide. Post-fixed sections were embedded in Durcupan ACM resin (cat# 44611-44614, MilliporeSigma) for 24 hours, placed between two ACLAR® embedding sheets (cat# 50425-25, Electron Microscopy Sciences) and resin was polymerised at 55°C for 72 hours. Region of interest –the barrel cortex– was excised, glued on a resin block, and cut into 75nm-thick ultrathin sections using a Leica Ultracut UC7 ultramicrotome (Leica Biosystems).

Ultrastructure characterisation

Dilation of the endoplasmic reticulum and Golgi apparatus cisternae was identified when the space between both cisternae membranes was greater than 50 nm[1]. Lysosomes were distinguished by their heterogenous electron-dense round structure, which was subdivided into primary, secondary (when associated with endosomes) and tertiary (when associated with lipidic inclusions and often with endosomes) categories[2,3]. Lipidic inclusions regrouped lipofuscins as well as lipidic bodies. Lipofuscins were recognised by their electron-dense spherical structure with a distinct fingerprint-like pattern, whereas lipid bodies showed a smooth and uniform pattern[3]. Mitochondrial elongation was identified by a length of 1 µm or greater[1].

Microglia were recognized by their immunoreactivity to Iba1 as well as ultrastructural features including: their dark irregular nuclei with a heterogenous chromatin pattern and dark irregular cytoplasm, often containing long stretch of endoplasmic reticulum cisternae and lipidic inclusions (i.e., lipofuscin, lipid bodies, lipid droplets and lysosomes)[4]. Neurons were distinguished by their pale nuclei and pale cytoplasm, often with an apical dendrite and synaptic contacts[4] (**Supplementary Figure 7f**). Synapses were identified by a visible synaptic density between the presynaptic axon terminal containing synaptic vesicles and the postsynaptic dendritic spine[4] (**Supplementary Figure 1c**). Astrocytic cells were identified by their pale nuclei with a thin rim of heterochromatin and their pale irregular cytoplasm, often containing intermediate filaments [4]. Glycogen granules were recognised as dark electron-dense granules[4] (**Supplementary Figure 7g**). Oligodendrocytes were distinguished by their dark round or oval nuclei with a heterogenous chromatin pattern and their dark squared-shape wide cytoplasm containing short, wide endoplasmic reticulum cisternae and often enriched in ribosomes[4] (**Supplementary Figure 7h**). Capillaries were counted when microglial cell bodies directly touched their basement membrane (**Supplementary Figure 1c**), which forms a thin electron-dense layer encompassing the capillary's cells including endothelial cells, pericytes and other perivascular cells (**Supplementary Figure 7a**). Digestive exophagy, also known as extracellular digestion, was identified by extracellular space pockets containing degraded elements or debris in areas directly adjacent to the microglial cell body[5–7] (**Supplementary Figure 7j**). Degraded myelin was recognized by ballooning, swelling or distancing between the well-defined myelin sheaths[4] and was often observed with exophagy (**Supplementary 7j**).

Behaviours.

For each behavioural test, animals were acclimated to the experimental room for 30 minutes prior to testing under the experimental lighting condition. All behaviours were assessed in a way to prevented mice to see the experimenter during testing. Between each trial and tests, arena and objects were thoroughly clean with ethanol/peroxide mixture to prevent any olfactory cues. Except for the open field, marbles burying and prepulse inhibition (PPI), all behavioural tests were filmed and scored automatically by the tracking software TopScan Version 2.00 (Clever Sys Inc, Reston, VA, United States), and scoring was verified by an observer blinded to experimental conditions.

Open field

Mice were placed in a transparent Plexiglas open field (40cm (width) x 40cm (length) x 30cm (height)) connected to VersaMax system (*Accuscan Instruments Inc*., Columbus, OH, United States) for 15 minutes after the dark phase (between 8:00 and 11:00). The test was divided into first 5 minutes of habituation and 10 minutes testing. VersaMax tracking system automatically recorded global locomotion parameters: speed, distance travelled, time spend in different areas of the arena (i.e., centre *versus* edge *versus* corner), horizontal and vertical movements, clockwise and anti-clockwise rotations, as well as stereotypic behaviours.

Novel Object Recognition

Novel object recognition consisted of a training and a testing phase in an opaque plexiglass box (40cm (width) x 40cm (length) x 30cm (height)) under the infrared light in the beginning of light phase (between 8:00 and 11:00). Mice were, first, let to explore two identical objects for 5 minutes during the acquisition phase. Then, the animals were put back in their home cage for 30 minutes. During the novel object recognition retention phase, mice were placed in the same experimental arena for 5 minutes in the presence of one of the two familiar objects along with a novel object of similar size. The novel object was randomly assigned. Object exploration was defined as touching or sniffing within the interaction zone (within ~2cm of the object). The recognition index was calculated by the ratio of the time spent exploring the object over

the total time allowed. Mice with an exploration time lower than 10 seconds per object were considered unsuitable and were not used.

Three-chambers social interaction

For two days prior to testing, stranger mice were habituated to the wired cage under infrared light for 20 minutes to minimise animal stress during social interaction assessment. Experimental mice were placed for 10 minutes in the central compartment and let free to explore the 3-chambers compartment (chamber: 26 cm (length) 21.6 (width) x 21.6 (height); door: 5 cm (width) x 5 cm (height)) with the two wired cages in the two side compartments. After the habituation to the three-chambers, a stranger mouse was placed in a cylindrical wired-cup (dimension: 7.6 cm (D) x 9.5 cm (H)) in one of the two side compartments and a toy in the other one. Social preference was assessed for 10 minutes, then the toy was substituted with a novel stranger to evaluate social novelty preference for another 10 minutes session. Time spent and number of entries in each compartment, as well as contacts/sniffing time with the occupied wired cage were measured using TopScan software. Social preference index and social novelty index were respectively calculated by the ratio of time interacting with stranger over toy, and time interacting with novel over familiar stranger.

Elevated plus maze

Elevated plus maze was assessed at the beginning of the light phase (8:00-11:00) under normal lighting (~30 lux). Mice were placed at the junction of the open and closed arms facing the open arms and let free to explore the elevated plus maze (open arm: 29.25 cm (length) x 5 cm (width); closed arm: 29.25 cm (length) x 5 cm (width) x 11.5 cm (height); centre: 5 cm (width) x 5 cm (length)) for 10 minutes. After the 5 minutes session, animal was put back in its home cage. Time spent and number of entries in open arms, closed arms and at the centre were measured to evaluate anxiety-related behaviour at basal level using TopScan software. Entry was considered when all three paws entered the arm.

Prepulse inhibition (PPI)

PPI is the inhibition of the startle response to a strong auditory stimulus when the stimulus is preceded by a weaker stimulus[8]. Alterations of the PPI are known to be commonly observed in neurodevelopmental disorders, we measured the sensorimotor gating which is altered in neurodevelopmental disorders, such as

schizophrenia[8]. Animals were placed in a cylindrical Plexiglas animal enclosure inside a commercially available system (SR-LAB; San Diego Instruments, San Diego, CA, United States). After 5 minutes of acclimation inside the enclosure, 42 discrete trials were performed; the first two trials are 120dB in magnitude followed by 40 trials in which the startle pulse was either alone or 100ms prior to a 30ms prepulse. The prepulses of intensity ranging from 3 to 15 dB were randomly presented to experimental mice. Startle responses were determined automatically by the SR-LAB system. PPI was calculated as the relative percentage of the mean amplitude of the startle response without prepulse compared to those recorded following a prepulse.

Supplementary figures

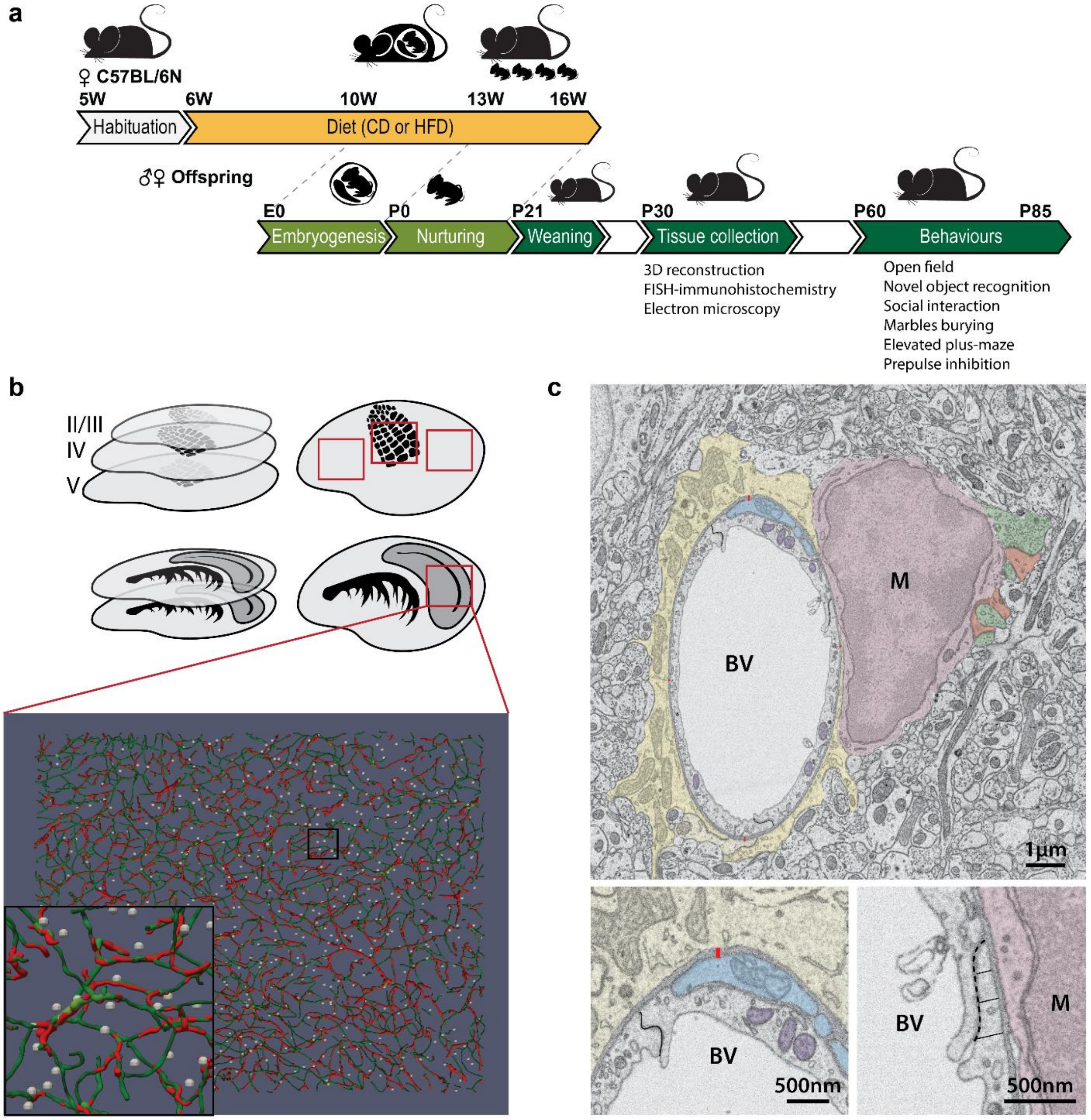

Supplementary Figure 1. Schematic of mHFD experimental design and neurovascular unit analysis. (a) Schematic illustrates diet protocol on the C57BL/6N dams (starting at 5 weeks old up to 16 weeks old, at weaning of their litter), as well as experimental protocol on offspring (mHFD exposure from E0 to P21, tissue collection at P30, and behavioural assessment from P60 to P85). (b) At P30, tissues were notably collected for analysis of the vascular unit organisation and structure *via* (b) 3D-reconstruction and (c) electron microscopy. (b) Flatten-brains were imaged on three sections of cortices (layer II/III, layer IV, layer

V of the anterior, parietal, and occipital cortices) and two sections of the CA1 hippocampus for 3D-reconstruction. The example of 3D-reconstruction shows endothelial cells in green, pericytes in red, and microglial cell bodies in white. (c) At nanoscale level, the ultrastructure of capillaries (identified by "BV") and microglia (identified by "M", pseudocoloured in fuchsia) were characterized. Number of astrocytic endfeet (pseudocoloured in yellow), pericytic processes (in light blue), tight junctions (highlighted by a fine black line) and mitochondria (in purple) were quantified. Tight junction length (dotted line) and thickness (fine grey lines) as well as basement membrane thickness (thick red lines) were measured. Similarly, microglial organelles and interactions with their microenvironment were analysed; here, we can see the microglia making a direct contact with the basement membrane of the capillary as well as several contacts with presynaptic axon terminals (pseudocoloured in green) and postsynaptic dendritic spines (pseudocoloured in red). ♀: female, ♂: male, CD: control diet, E: embryonic day, HFD: maternal high-fat diet, P: postnatal day, W: week.

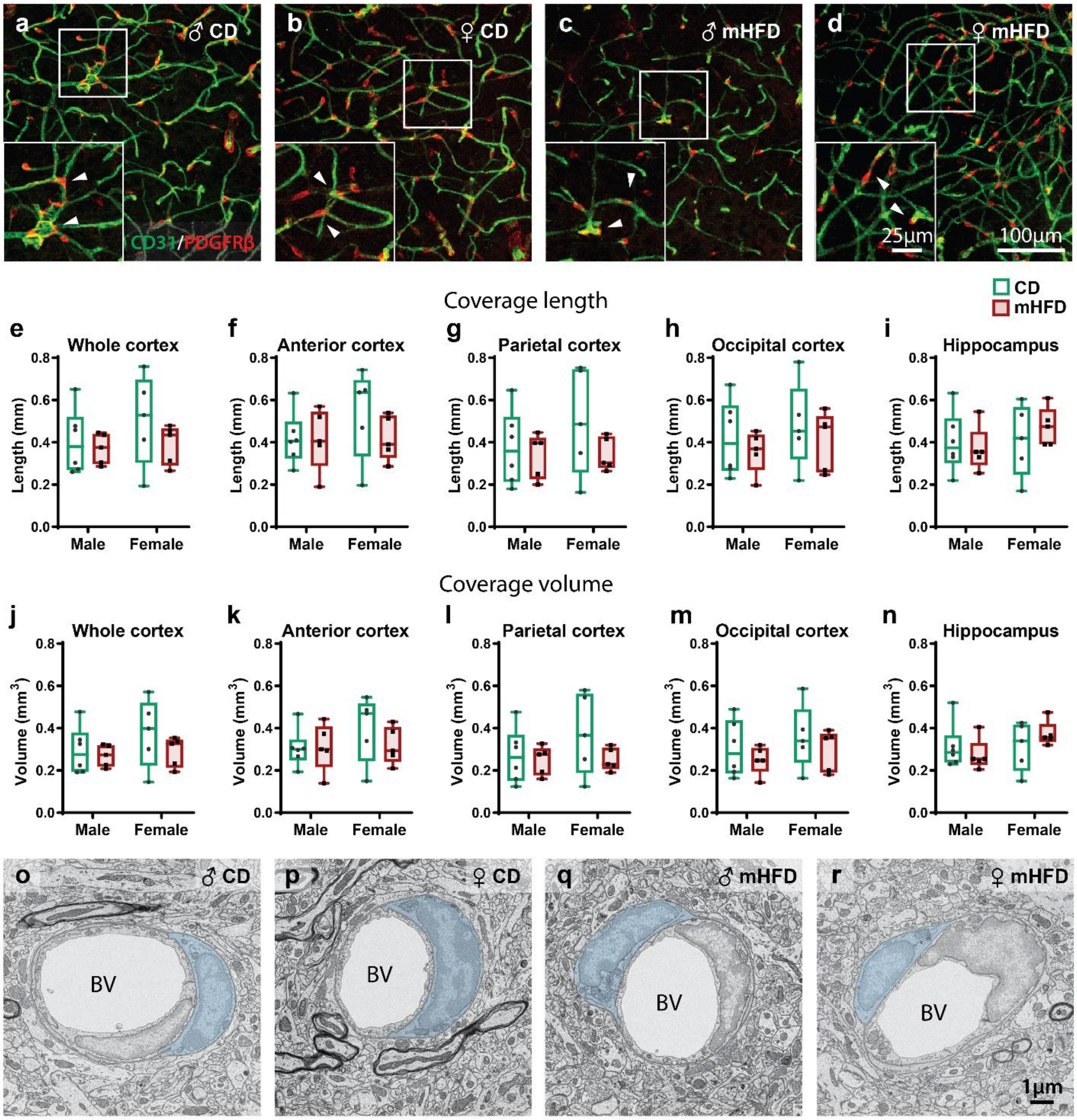

Supplementary Figure 2. mHFD had no significant effect on cortical and hippocampal pericyte coverage in P30 male and female offspring. (a-d) Pictures at 20x with an epifluoresence microscope illustrate pericyte coverage of the blood vessels from the parietal cortex of adolescent offspring, where white arrows indicate colocalisation on the inset. (e-i) Pericyte coverage length and (j-n) coverage volume were measured for the (e, j) whole cortex, (f, k) anterior cortex, (g, l) parietal cortex, (h, m) occipital cortex and (i, n) hippocampus of the adolescent offspring (n=5-6 animals/diet/sex). (o-r) At the ultrastructural level,

pericytes (pseudocoloured in light blue) are well anchored to the capillary (identified “BV”) (n=93-95 capillaries/diet/sex, N=4 animals). Whisker graphs show minimum, median (line) and maximum, as well as individual data points. ♀: female, ♂: male, CD: control diet, mHFD: maternal high-fat diet.

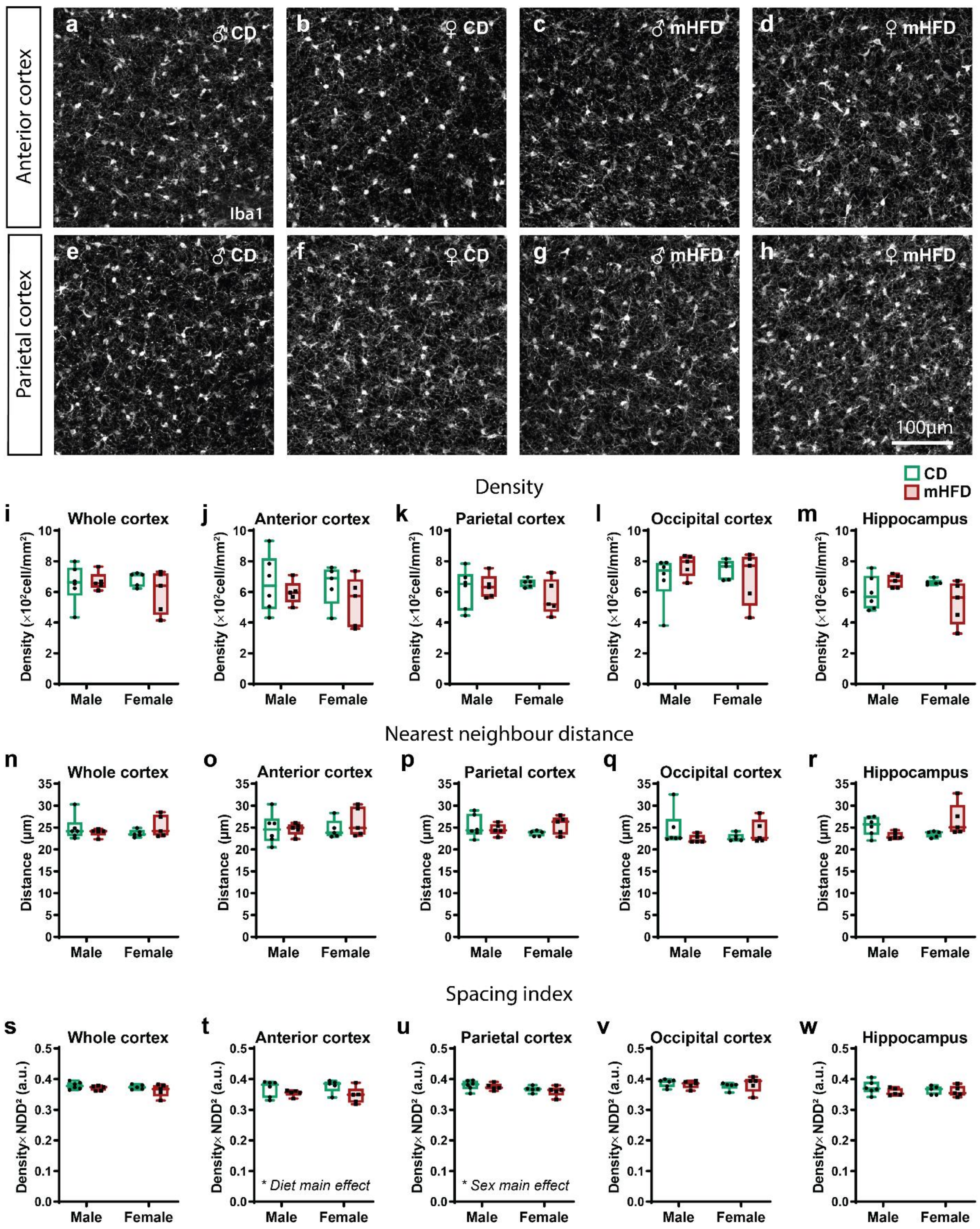

Supplementary Figure 3. The effect of mHFD on cortical and hippocampal microglial cell density, nearest neighbour distance and spacing distance in male and female P30 offspring. (a-h) Pictures taken

at 20x with an epifluorescence microscope illustrate Iba1^{+} microglia (in white) cell density and their distribution from the (a-d) anterior cortex and (e-h) parietal cortex of adolescent offspring. (i-m) Density, (n-r) nearest neighbour distance and (s-w) spacing index were determined for the (i, n, s) whole cortex, (j, o, t) anterior cortex, (k, p, u) parietal cortex, (l, q, v) occipital cortex and (m, r, w) hippocampus of the adolescent offspring. Whisker graphs show minimum, median (black line) and maximum, as well as individual data points (n=5-6 animals/diet/sex). *P<0.05 by mixed-effect analysis identifying a sex effect. ♀: female, ♂: male, a.u.: arbitrary unit, CD: control diet, mHFD: maternal high-fat diet NND: nearest neighbour distance.

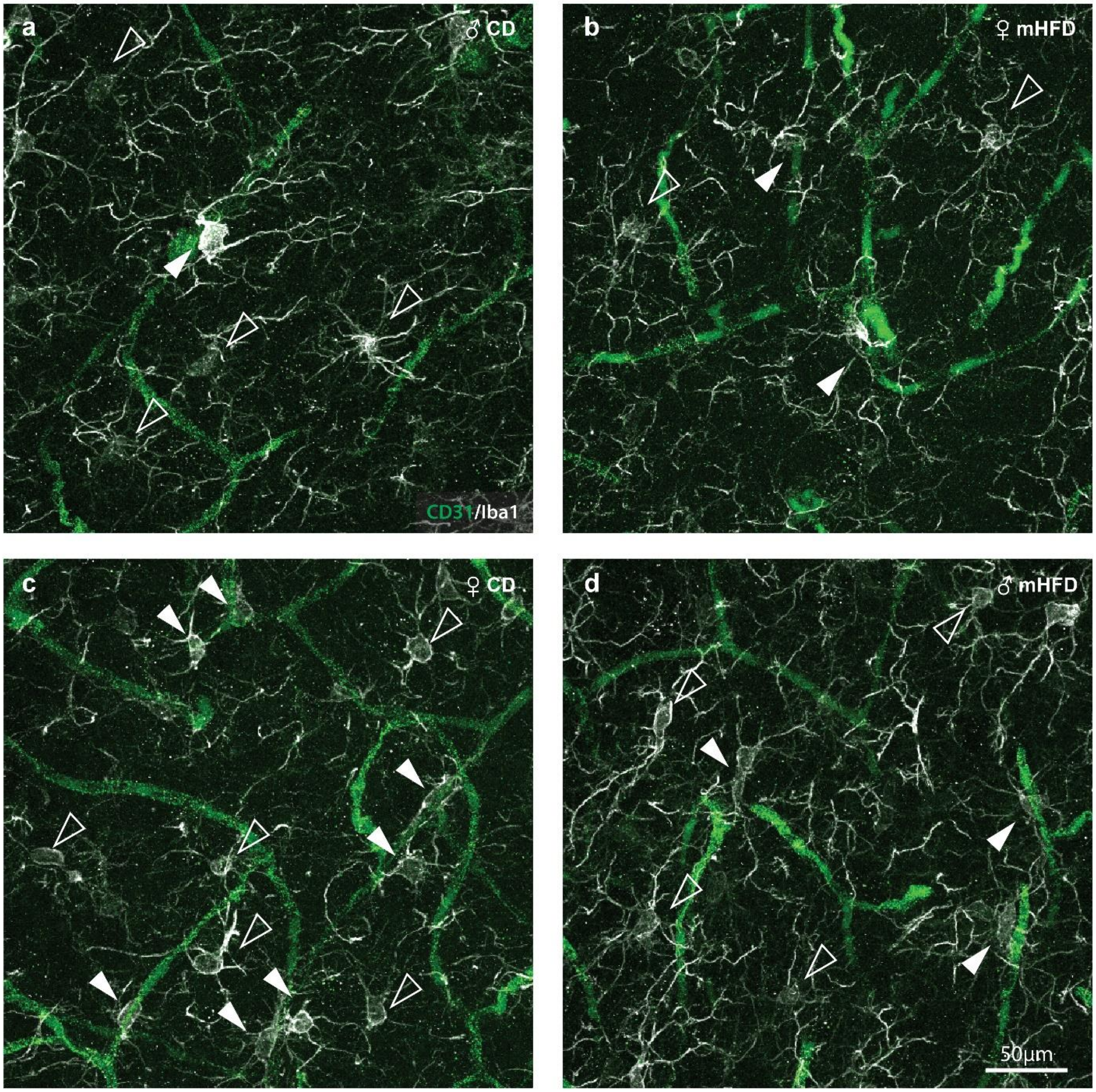

Supplementary Figure 4. Effects of mHFD on microglial distance to blood vessels and their putative contacts with blood vessels in CA1 hippocampus of P30 offspring. (a-d) Pictures taken at 63x by a confocal microscope show microglia interaction with blood vessels from the CA1 hippocampus, where microglia making putative contacts (filled white arrowheads) and microglia that are not making putative contacts (open white arrowheads) are identified. ♀: female, ♂: male, CD: control diet, mHFD: maternal high-fat diet.

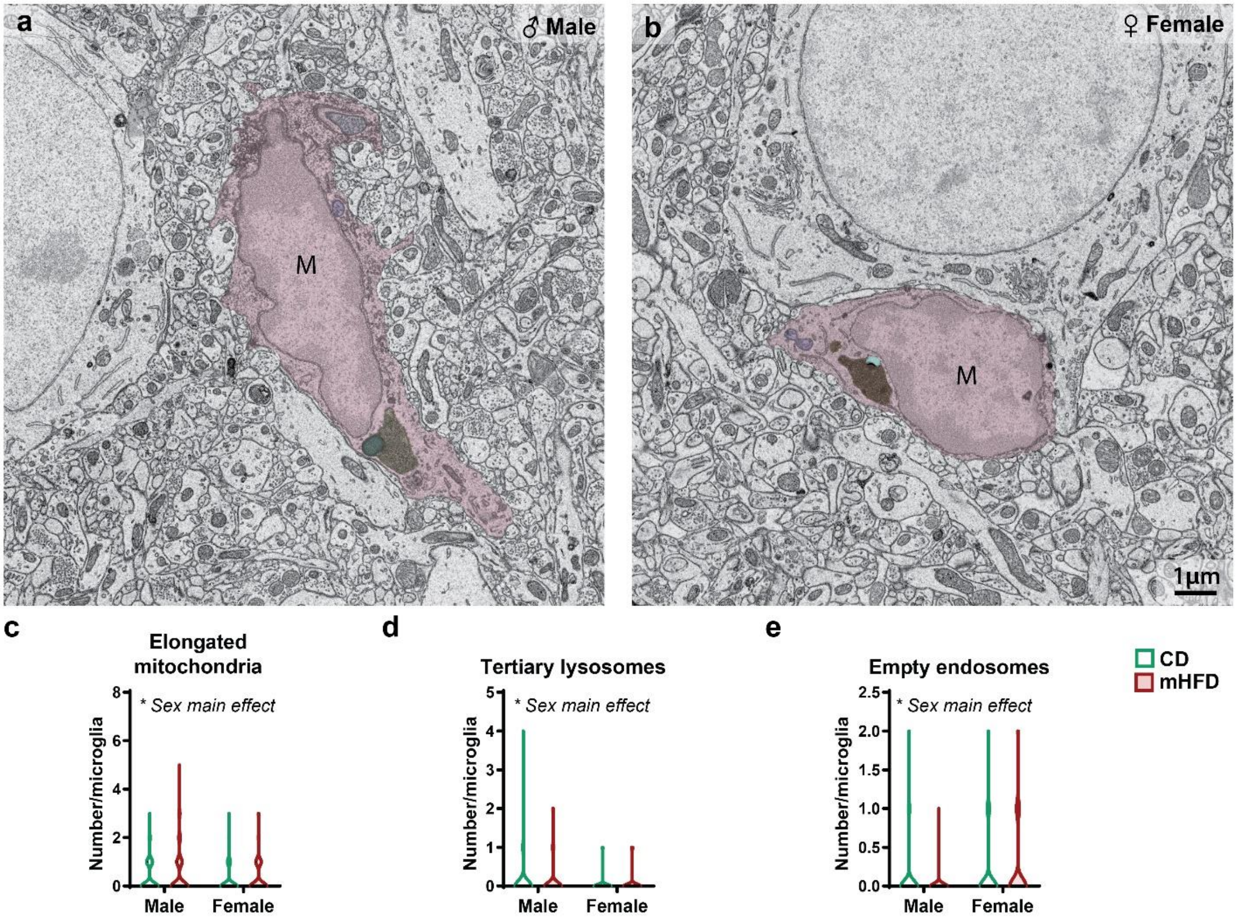

Supplementary Figure 5. Microglia from the parietal cortex exhibit ultrastructural sex differences. (a-b) Micrographs illustrate main ultrastructural features of microglia (identified by "M", pseudocoloured in fuchsia) presenting sex differences regardless of their maternal diet, in which mitochondria are pseudocoloured in purple, elongated mitochondria in light purple, tertiary lysosomes in orange, primary and secondary lysosomes in dark orange, lipid body in turquoise and empty endosome in teal. Notably, microglia from male offspring cortex had an increased number of (c) elongated mitochondria, (d) tertiary lysosomes and (e) decreased number of empty endosomes. Violin graphs show minimum, median (black line) and maximum. $^{\#}P<0.05$ by mixed-effect analysis identifying a sex effect. ♀: female, ♂: male, CD: control diet, mHFD: maternal high-fat diet.

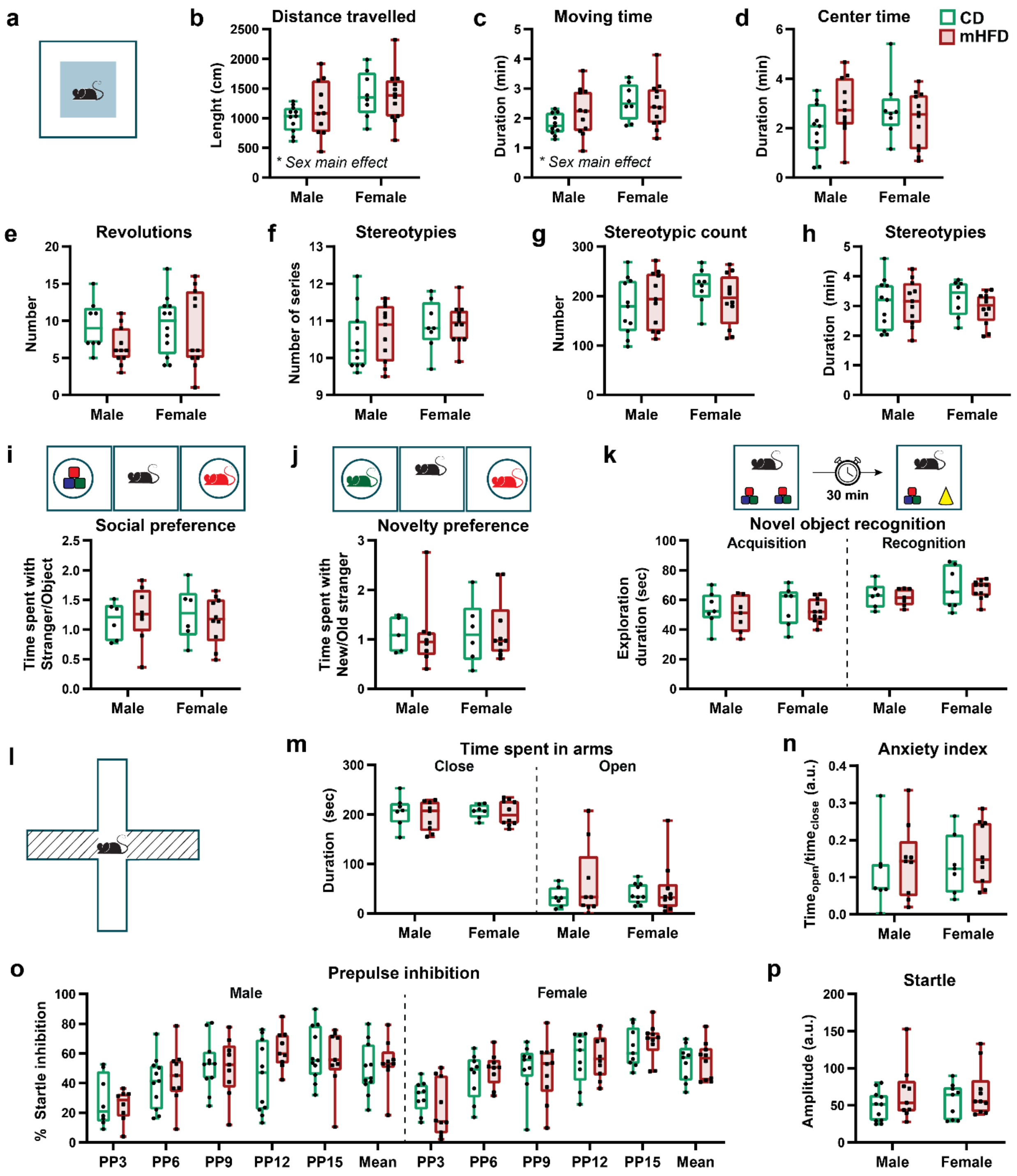

Supplementary Figure 6. The effect of mHFD on motor function, spatial memory, social interaction, anxiety-like behaviours, and sensorimotor gating in adult male and female adult offspring. Schemes of the experimental paradigm of (a) open field, (i) elevated plus-maze, (l-m) three-chambers test and (n) novel object recognition test are illustrated. (b) Distance travelled, (c) moving time, (d) time spent in the

centre, (e) number of revolutions, (f) stereotypies, (g) stereotypic count and (h) stereotypies number were measured by open field to assess global motor function. (k) Anxiety index was calculated with (j) time spent in arms during elevated plus-maze test. (l) Social preference and (m) novelty preference were evaluated using three-chambers test. (n) Spatial working memory was appraised by novel object recognition. (o) Sensorimotor gating of the acoustic startle response and (p) acoustic startle response were measured by the prepulse inhibition test. Whisker graph shows minimum, median (line) and maximum, as well as individual data points (n=6-10 litters/diet/sex). *P<0.05 by 2-way ANOVA analysis : female, ♂: male, a.u.: arbitrary unit, CD: control diet, mHFD: maternal high-fat diet.

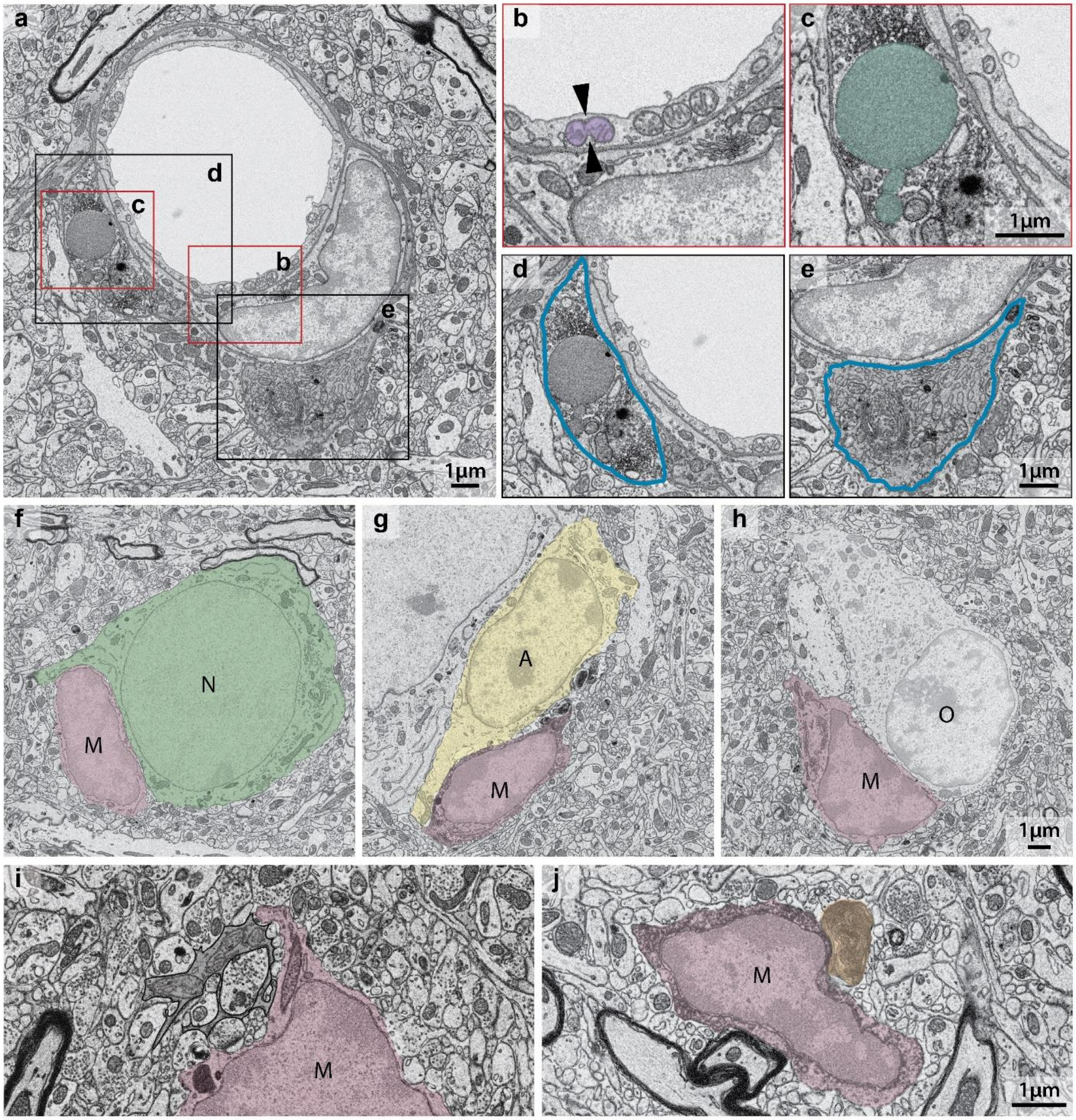

Supplementary Figure 7. Examples of other ultrastructural parameters analysed. (a) A lower magnification of a capillary in which we can observe (b) fusion or fission of mitochondria pseudocoloured in purple, (c) lipid bodies pseudocoloured in turquoise as well as (d) Iba1+ cells (edge of the cell trace in blue) and (e) other undistinguished perivascular cells (edge of the cell trace in blue) within the perivascular space of capillaries. (f-i) Following panels illustrate microglial interactions with other cells of the brains, including (f) neuron (identified "N", pseudocoloured in green), (g) astrocyte (identified "A",

pseudocoloured in yellow) and (h) oligodendrocyte (identified “O”, pseudocoloured in white), as well as (i) darker cell processes (edge of the process are highlighted by a black line), and (j) degraded myelin (pseudocoloured in orange). For each panel, microglia are pseudocoloured in fuchsia and identified by a “M” when visible.

Supplementary tables

Supplementary Table 1. Summary of ANOVAs test to assess *Diet* and *Sex* main effects as well as *Diet*Sex* interaction effect of 2D and 3D analyses of the neurovascular unit data. Data are presented as Mean ± SEM for each treatment group. *F* and *P* values are given for main and interactions effects (n=5-6 animals/diet/sex, 2-3 sections). ♂: male, ♀: female, a.u.: arbitrary unit, CD: control diet, mHFD: maternal high-fat diet, SEM: standard error of the mean.

Parameters			Mean ± SEM	F	P
Whole cortex	Blood vessels	Vessel length (mm/mm^3)	♂-CD: 1.672 ± 0.048<br>♂-mHFD: 1.997 ± 0.047<br>♀-CD: 1.647 ± 0.088<br>♀-mHFD: 1.798 ± 0.067	Diet*Sex: 1.900<br>Sex: 3.095<br>Diet: 14.06	Diet*Sex: 0.1860<br>Sex: 0.0965<br>Diet: 0.0016 **
		Number of branching points ($/mm^3$)	♂-CD: 2.950 ± 0.161<br>♂-mHFD: 4.040 ± 0.179<br>♀-CD: 3.393 ± 0.217<br>♀-mHFD: 4.033 ± 0.265	Diet*Sex: 1.193<br>Sex: 1.122<br>Diet: 17.63	Diet*Sex: 0.2900<br>Sex: 0.3044<br>Diet: 0.0006***
		Tortuosity index (a.u.)	♂-CD: 1.791 ± 0.027<br>♂-mHFD: 1.832 ± 0.027<br>♀-CD: 1.784 ± 0.011<br>♀-mHFD: 1.799 ± 0.018	Diet*Sex: 0.3285<br>Sex: 0.7602<br>Diet: 1.550	Diet*Sex: 0.5740<br>Sex: 0.3954<br>Diet: 0.2301
	Pericytes	Length coverage (mm)	♂-CD: 0.404 ± 0.063<br>♂-mHFD: 0.370 ± 0.033<br>♀-CD: 0.506 ± 0.097<br>♀-mHFD: 0.390 ± 0.042	Diet*Sex: 0.4108<br>Sex: 0.9169<br>Diet: 1.381	Diet*Sex: 0.5301<br>Sex: 0.3517<br>Diet: 0.2562
		Volume coverage	♂-CD: 0.293 ± 0.046<br>♂-mHFD: 0.269 ± 0.023<br>♀-CD: 0.377 ± 0.073<br>♀-mHFD: 0.289 ± 0.032	Diet*Sex: 0.4603<br>Sex: 1.189<br>Diet: 1.393	Diet*Sex: 0.5066<br>Sex: 0.2907<br>Diet: 0.2542
	Microglia	Microglia density ratio (a.u.)	♂-CD: 0.929 ± 0.132<br>♂-mHFD: 1.052 1 0.044<br>♀-CD: 1.085 ± 0.061<br>♀-mHFD: 1.040 ± 0.108	Diet*Sex: 0.7117<br>Sex: 0.5263<br>Diet: 0.1563	Diet*Sex: 0.4106<br>Sex: 0.4780<br>Diet: 0.6975
		Microglial nearest distance to blood vessel (µm)	♂-CD: 6.676 ± 0.253<br>♂-mHFD: 5.853 ± 0.114<br>♀-CD: 5.764 ± 0.216<br>♀-mHFD: 5.282 ± 0.086	Diet*Sex: 0.7873<br>Sex: 14.94<br>Diet: 11.57	Diet*Sex: 0.3873<br>Sex: 0.0012**<br>Diet: 0.0034**
		Ratio microglia in contact with blood vessel (a.u.)	♂-CD: 0.473 ± 0.016<br>♂-mHFD: 0.520 ± 0.007<br>♀-CD: 0.529 ± 0.017	Diet*Sex: 0.1134<br>Sex: 15.88<br>Diet: 10.57	Diet*Sex: 0.7405<br>Sex: 0.0010***<br>Diet: 0.0047**

			♀-mHFD: 0.567 ± 0.008		
		Microglial density (cell/mm^2)	♂-CD: 6.542 ± 0.513<br>♂-mHFD: 6.682 ± 0.260<br>♀-CD: 6.829 ± 0.205<br>♀-mHFD: 5.972 ± 0.628	*Diet*Sex*: 1.228<br>*Sex*: 0.2204<br>*Diet*: 0.6360	*Diet*Sex*: 0.2833<br>*Sex*: 0.6447<br>*Diet*: 0.4361
		NND (mm)	♂-CD: 24.86 ± 1.13<br>♂-mHFD: 23.88 ± 0.41<br>♀-CD: 23.66 ± 0.37<br>♀-mHFD: 25.29 ± 1.13	*Diet*Sex*: 2.155<br>*Sex*: 0.01373<br>*Diet*: 0.1312	*Diet*Sex*: 0.1603<br>*Sex*: 0.9081<br>*Diet*: 0.7217
		Spacing index (a.u.)	♂-CD: 0.379 ± 0.005<br>♂-mHFD: 0.370 ± 0.004<br>♀-CD: 0.374 ± 0.004<br>♀-mHFD: 0.363 ± 0.009	*Diet*Sex*: 0.03944<br>*Sex*: 1.095<br>*Diet*: 2.705	*Diet*Sex*: 0.8449<br>*Sex*: 0.3100<br>*Diet*: 0.1184
Anterior cortex	Blood vessels	Vessel length (mm/mm^3)	♂-CD: 1.689 ± 0.072<br>♂-mHFD: 2.017 ± 0.065<br>♀-CD: 1.697 ± 0.142<br>♀-mHFD: 1.849 ± 0.109	*Diet*Sex*:0.7908<br>*Sex*:0.6465<br>*Diet*:5.804	*Diet*Sex*:0.3863<br>*Sex*:0.4325<br>*Diet*:**0.0276***
		Number of branching points (/mm^3)	♂-CD: 3.155 ± 0.254<br>♂-mHFD: 4.425 ± 0.332<br>♀-CD: 3.661 ± 0.416<br>♀-mHFD: 4.229 ± 0.387	Diet*Sex*: 1.032<br>*Sex*: 0.2015<br>*Diet*: 7.083	*Diet*Sex*: 0.3238<br>*Sex*: 0.6592<br>*Diet*: **0.0164***
		Tortuosity index (a.u.)	♂-CD: 1.810 ± 0.039<br>♂-mHFD: 1.839 ± 0.046<br>♀-CD: 1.791 ± 0.047<br>♀-mHFD: 1.823 ± 0.026	*Diet*Sex*: 0.001931<br>*Sex*: 0.6659<br>*Diet*: 0.4547	*Diet*Sex*: 0.9655<br>*Sex*:0.6659<br>*Diet*:0.4547
	Pericytes	Length coverage (mm)	♂-CD: 0.418 ± 0.050<br>♂-mHFD: 0.415 ± 0.066<br>♀-CD: 0.539 ± 0.096<br>♀-mHFD: 0.419 ± 0.047	*Diet*Sex*: 0.7560<br>*Sex*: 0.8830<br>*Diet*: 0.8549	*Diet*Sex*: 0.3967<br>*Sex*: 0.3605<br>*Diet*: 0.3681
		Volume coverage	♂-CD: 0.306 ± 0.037<br>♂-mHFD: 0.310 ± 0.051<br>♀-CD: 0.399 ± 0.071<br>♀-mHFD: 0.318 ± 0.039	*Diet*Sex*: 0.7225<br>*Sex*: 1.020<br>*Diet*: 0.5795	*Diet*Sex*: 0.4071<br>*Sex*: 0.3267<br>*Diet*: 0.4569
	Microglia	Microglia density ratio (a.u.)	♂-CD: 0.954 ± 1.55<br>♂-mHFD: 1.053 ± 0.063<br>♀-CD: 1.055 ± 0.112<br>♀-mHFD: 0.920 ± 0.139	*Diet*Sex*: 0.8436<br>*Sex*: 0.01526<br>*Diet*: 0.02011	*Diet*Sex*: 0.3712<br>*Sex*: 0.9031<br>*Diet*: 0.8889
		Microglial nearest distance to blood vessel (µm)	♂-CD: 6.672 ± 0.275<br>♂-mHFD: 5.487 ± 0.180<br>♀-CD: 5.647 ± 0.159<br>♀-mHFD: 5.247 ± 0.111	*Diet*Sex*: 3.720<br>*Sex*: 9.655<br>*Diet*: 15.16	*Diet*Sex*: 0.0706<br>*Sex*: **0.0064 ****<br>*Diet*: **0.0012****
			♂-CD: 0.477 ± 0.016<br>♂-mHFD: 0.549 ± 0.013	*Diet*Sex*: 2.490<br>*Sex*: 9.325	*Diet*Sex*: 0.1330<br>*Sex*: **0.0072****

		Ratio microglia in contact with blood vessel (a.u.)	♀-CD: 0.540 ± 0.015<br>♀-mHFD: 0.569 ± 0.008	*Diet*: 14.00	*Diet*: **0.0016****
		Microglial density (cell/mm^2)	♂-CD: 6.560 ± 0.765<br>♂-mHFD: 5.955 ± 0.343<br>♀-CD: 6.454 ± 0.596<br>♀-mHFD: 5.354 ± 0.729	*Diet*Sex*: 0.1449<br>*Sex*: 0.2945<br>*Diet*: 1.712	*Diet*Sex*: 0.7082<br>*Sex*: 0.5944<br>*Diet*: 0.2082
		NND (mm)	♂-CD: 24.70 ± 1.43<br>♂-mHFD: 24.66 ± 0.63<br>♀-CD: 24.61 ± 0.98<br>♀-mHFD: 26.25 ± 1.49	*Diet*Sex*: 0.4768<br>*Sex*: 0.3725<br>*Diet*: 0.4323	*Diet*Sex*: 0.4992<br>*Sex*: 0.5497<br>*Diet*: 0.5196
		Spacing index (a.u.)	♂-CD: 0.369 ± 0.011<br>♂-mHFD: 0.354 ± 0.005<br>♀-CD: 0.377 ± 0.010<br>♀-mHFD: 0.347 ± 0.012	*Diet*Sex*: 0.6695<br>*Sex*: 0.0006887<br>*Diet*: 5.341	*Diet*Sex*: 0.4245<br>*Sex*:0.9794<br>*Diet*: **0.0336***
Parietal cortex	Blood vessels	Vessel length (mm/mm^3)	♂-CD: 1.837 ± 0.040<br>♂-mHFD: 2.128 ± 0.024<br>♀-CD: 1.774 ± 0.072<br>♀-mHFD: 1.990 ± 0.065	*Diet*Sex*: 0.4950<br>*Sex*: 3.676<br>*Diet*: 23.23	*Diet*Sex*:0.4912<br>*Sex*: 0.0722<br>*Diet*: **0.0002*****
		Number of branching points (/mm^3)	♂-CD: 3.225 ± 0.233<br>♂-mHFD: 4.258 ± 0.144<br>♀-CD: 3.705 ± 0.202<br>♀-mHFD: 4.701 ± 0.309	*Diet*Sex*: 0.006343<br>*Sex*: 3.954<br>*Diet*: 19.13	*Diet*Sex*: 0.9374<br>*Sex*: 0.0631<br>*Diet*:**0.0004*****
		Tortuosity index (a.u.)	♂-CD: 1.809 ± 0.053<br>♂-mHFD: 1.866 ± 0.027<br>♀-CD: 1.789 ± 0.003<br>♀-mHFD: 1.745 ± 0.033	*Diet*Sex*: 1.811<br>*Sex*: 3.609<br>*Diet*: 0.02876	*Diet*Sex*: 0.1961<br>*Sex*: 0.0746<br>*Diet*: 0.8673
	Pericytes	Length coverage (mm)	♂-CD: 0.374 ± 0.072<br>♂-mHFD: 0.338 ± 0.048<br>♀-CD: 0.499 ± 0.114<br>♀-mHFD: 0.343 ± 0.036	*Diet*Sex*: 0.6472<br>*Sex*: 0.7686<br>*Diet*: 1.684	*Diet*Sex*: 0.4322<br>*Sex*: 0.3929<br>*Diet*: 0.2117
		Volume coverage	♂-CD: 0.269 ± 0.053<br>♂-mHFD: 0.248 ± 0.031<br>♀-CD: 0.374 ± 0.087<br>♀-mHFD: 0.252 ± 0.024	*Diet*Sex*: 0.8442<br>*Sex*: 0.9838<br>*Diet*: 1.718	*Diet*Sex*: 0.3711<br>*Sex*: 0.3352<br>*Diet*: 0.2074
	Microglia	Microglia density ratio (a.u.)	♂-CD: 0.924 ± 0.140<br>♂-mHFD: 1.003 ± 0.058<br>♀-CD: 1.091 ± 0.055<br>♀-mHFD: 1.108 ± 0.095	*Diet*Sex*: 0.09519<br>*Sex*: 1.841<br>*Diet*: 0.2311	*Diet*Sex*: 0.7614<br>*Sex*: 0.1925<br>*Diet*: 0.6369
		Microglial nearest distance to blood vessel (µm)	♂-CD: 5.967 ± 0.212<br>♂-mHFD: 5.433 ± 0.178<br>♀-CD: 5.267 ± 0.263<br>♀-mHFD: 4.700 ± 0.152	*Diet*Sex*: 0.006442<br>*Sex*: 11.91<br>*Diet*: 7.016	*Diet*Sex*: 0.9370<br>*Sex*: **0.0030****<br>*Diet*: **0.0169***
			♂-CD: 0.509 ± 0.019	*Diet*Sex*: 0.1783	*Diet*Sex*: 0.6781

		Ratio microglia in contact with blood vessel (a.u.)	♂-mHFD: 0.547 ± 0.015<br>♀-CD: 0.555 ± 0.024<br>♀-mHFD: 0.608 ± 0.013	*Sex*: 8.595<br>*Diet*: 6.041	*Sex*: **0.0093****<br>*Diet*: **0.0250***
		Microglial density (cell/mm^2)	♂-CD: 6.199 ± 0.524<br>♂-mHFD: 6.348 ± 0.345<br>♀-CD: 6.583 ± 0.123<br>♀-mHFD: 5.659 ± 0.517	*Diet***Sex*: 1.572<br>*Sex*: 0.1273<br>*Diet*: 0.8218	*Diet***Sex*: 0.2268<br>*Sex*: 0.7257<br>*Diet*: 0.3773
		NND (mm)	♂-CD: 25.25 ± 1.06<br>♂-mHFD: 24.59 ± 0.58<br>♀-CD: 23.66 ± 0.29<br>♀-mHFD: 25.56 ± 0.92	*Diet***Sex*: 2.453<br>*Sex*: 0.1451<br>*Diet*: 0.5775	*Diet***Sex*: 0.1357<br>*Sex*: 0.7080<br>*Diet*: 0.4577
		Spacing index (a.u.)	♂-CD: 0.380 ± 0.007<br>♂-mHFD: 0.375 ± 0.005<br>♀-CD: 0.367 ± 0.005<br>♀-mHFD: 0.361 ± 0.007	*Diet***Sex*: 0.009025<br>*Sex*: 4.948<br>*Diet*: 0.9504	*Diet***Sex*: 0.9254<br>*Sex*: **0.0399***<br>*Diet*: 0.3433
Occipital cortex	Blood vessels	Vessel length (mm/mm^3)	♂-CD: 1.489 ± 0.094<br>♂-mHFD: 1.846 ± 0.083<br>♀-CD: 1.472 ± 0.077<br>♀-mHFD: 1.555 ± 0.046	*Diet***Sex*: 2.959<br>*Sex*: 3.746<br>*Diet*: 7.681	*Diet***Sex*: 0.1036<br>*Sex*: 0.0697<br>*Diet*: **0.0131***
		Number of branching points (/mm^3)	♂-CD: 2.470 ± 0.278<br>♂-mHFD: 3.437 ± 0.218<br>♀-CD: 2.814 ± 0.187<br>♀-mHFD: 3.170 ± 0.216	*Diet***Sex*: 1.689<br>*Sex*: 0.02679<br>*Diet*: 7.929	*Diet***Sex*: 0.2110<br>*Sex*: 0.8719<br>*Diet*: **0.0119***
		Tortuosity index (a.u.)	♂-CD: 1.754 ± 0.040<br>♂-mHFD: 1.791 ± 0.059<br>♀-CD: 1.774 ± 0.031<br>♀-mHFD: 1.830 ± 0.052	*Diet***Sex*: 0.04131<br>*Sex*:0.4022<br>*Diet*:1.018	*Diet***Sex*: 0.8414<br>*Sex*: 0.5344<br>*Diet*: 0.3271
	Pericytes	Length coverage (mm)	♂-CD: 0.419 ± 0.073<br>♂-mHFD: 0.357 ± 0.044<br>♀-CD: 0.481 ± 0.091<br>♀-mHFD: 0.407 ± 0.063	*Diet***Sex*: 0.006198<br>*Sex*: 0.6348<br>*Diet*: 0.9225	*Diet***Sex*: 0.9382<br>*Sex*: 0.4366<br>*Diet*: 0.3503
		Volume coverage	♂-CD: 0.304 ± 0.054<br>♂-mHFD: 0.250 ± 0.030<br>♀-CD: 0.359 ± 0.069<br>♀-mHFD: 0.297 ± 0.044	*Diet***Sex*: 0.005284<br>*Sex*: 0.9421<br>*Diet*: 1.255	*Diet***Sex*: 0.9429<br>*Sex*: 0.3453<br>*Diet*: 0.2782
	Microglia	Microglia density ratio (a.u.)	♂-CD: 0.911 ± 0.149<br>♂-mHFD: 1.095 ± 0.077<br>♀-CD: 1.106 ± 0.053<br>♀-mHFD: 1.092 ± 0.115	*Diet***Sex*: 0.7910<br>*Sex*: 0.7425<br>*Diet*: 0.5711	*Diet***Sex*: 0.3862<br>*Sex*: 0.4008<br>*Diet*: 0.4602
		Microglial nearest distance to blood vessel (µm)	♂-CD: 7.389 ± 0.384<br>♂-mHFD: 6.640 ± 0.137<br>♀-CD: 6.380 ± 0.359<br>♀-mHFD: 5.900 ± 0.136	*Diet***Sex:* 0.2072<br>*Sex*: 8.763<br>*Diet*: 4.327	*Diet***Sex*: 0.6548<br>*Sex*: **0.0088****<br>*Diet*: 0.0530

		Ratio microglia in contact with blood vessel (a.u.)	♂-CD: 0.432 ± 0.020<br>♂-mHFD: 0.463 ± 0.006<br>♀-CD: 0.492 ± 0.021<br>♀-mHFD: 0.524 ± 0.016	*Diet***Sex*: 0.0008377<br>*Sex*: 12.40<br>*Diet*: 3.325	*Diet***Sex*:0.9772<br>*Sex*: **0.0026****<br>*Diet*: 0.0859
		Microglial density (cell/mm^2)	♂-CD: 6.867 ± 0.638<br>♂-mHFD: 7.742 ± 0.328<br>♀-CD: 7.451 ± 0.284<br>♀-mHFD: 6.903 ± 0.783	*Diet***Sex*: 1.590<br>*Sex*: 0.05123<br>*Diet*: 0.08403	*Diet***Sex:* 0.2244<br>*Sex*: 0.8236<br>*Diet*: 0.7754
		NND (mm)	♂-CD: 24.63 ± 1.64<br>♂-mHFD: 22.38 ± 0.42<br>♀-CD: 22.72 ± 0.40<br>♀-mHFD: 24.04 ± 1.23	*Diet***Sex*: 2.414<br>*Sex*: 0.01126<br>*Diet*: 0.1599	*Diet***Sex*: 0.1387<br>*Sex*: 0.9167<br>*Diet*: 0.6942
		Spacing index (a.u.)	♂-CD: 0.381 ± 0.005<br>♂-mHFD: 0.382 ± 0.006<br>♀-CD: 0.377 ± 0.005<br>♀-mHFD: 0.383 ± 0.012	*Diet***Sex*: 0.4983<br>*Sex*: 0.4345<br>*Diet*: 0.0005275	*Diet***Sex*: 0.4898<br>*Sex*: 0.5186<br>*Diet*: 0.9819
Hippocampus	Blood vessels	Vessel length (mm/mm^3)	♂-CD: 1.383 ± 1.102<br>♂-mHFD: 1.646 ± 0.061<br>♀-CD: 1.518 ± 0.205<br>♀-mHFD: 1.275 ± 0.042	*Diet***Sex*: 4.480<br>*Sex*: 0.9751<br>*Diet*: 0.007500	*Diet***Sex*: **0.0493***<br>*Sex*: 0.3373<br>*Diet*: 0.9320
		Number of branching points (/mm^3)	♂-CD: 2.238 ± 0.245<br>♂-mHFD: 3.178 ± 0.270<br>♀-CD: 2.886 ± 0.516<br>♀-mHFD: 2.236 ± 0.169	*Diet***Sex*: 6.118<br>*Sex*: 0.2087<br>*Diet*: 0.2047	*Diet***Sex*: **0.0242***<br>*Sex*: 0.6536<br>*Diet*: 0.6567
		Tortuosity index (a.u.)	♂-CD: 1.800 ± 0.030<br>♂-mHFD: 1.958 ± 0.037<br>♀-CD: 1.848 ± 0.056<br>♀-mHFD: 1.856 ± 0.075	*Diet***Sex*: 2.145<br>*Sex*: 0.2770<br>*Diet*: 2.664	*Diet***Sex*: 0.1613<br>*Sex*: 0.6055<br>*Diet*: 0.1210
	Pericytes	Length coverage (mm)	♂-CD: 0.401 ± 0.058<br>♂-mHFD: 0.368 ± 0.048<br>♀-CD: 0.410 ± 0.077<br>♀-mHFD: 0.474 ± 0.041	*Diet***Sex*: 0.7118<br>*Sex*: 0.9734<br>*Diet*: 0.07137	*Diet***Sex*: 0.4106<br>*Sex*: 0.3377<br>*Diet*: 0.7926
		Volume coverage	♂-CD: 0.313 ± 0.044<br>♂-mHFD: 0.273 ± 0.034<br>♀-CD: 0.314 ± 0.052<br>♀-mHFD: 0.373 ± 0.027	*Diet***Sex*: 1.443<br>*Sex*: 1.553<br>*Diet*: 0.05501	*Diet***Sex*: 0.2461<br>*Sex*: 0.2296<br>*Diet*: 0.8174
	Microglia	Microglia density ratio (a.u.)	♂-CD: 0.836 ± 0.099<br>♂-mHFD: 1.064 ± 0.023<br>♀-CD: 1.185 ± 0.046<br>♀-mHFD: 0.932 ± 0.153	*Diet***Sex*: 6.315<br>*Sex*: 1.294<br>*Diet*: 0.01648	*Diet***Sex*: **0.0223***<br>*Sex*:0.2711<br>*Diet*: 0.8994
		Microglial nearest distance to blood vessel (µm)	♂-CD: 8.900 ± 0.313<br>♂-mHFD: 7.650 ± 0.439<br>♀-CD: 7.067 ± 0.435	*Diet***Sex*: 5.674<br>*Sex*: 5.799<br>*Diet*: 0.7814	*Diet***Sex*: **0.0292***<br>*Sex*: **0.0277***<br>*Diet*:0.3890

			♀-mHFD: 7.640 ± 0.346		
		Ratio microglia in contact with blood vessel (a.u.)	♂-CD: 0.397 ± 0.017<br>♂-mHFD: 0.460 ± 0.019<br>♀-CD: 0.481 ± 0.020<br>♀-mHFD: 0.462 ± 0.018	*Diet*Sex*: 4.878<br>*Sex*: 5.361<br>*Diet*: 1.382	*Diet*Sex*: **0.0412***<br>*Sex*: **0.0333***<br>*Diet*: 0.2560
		Microglial density (cell/mm^2)	♂-CD: 5.925 ± 0.453<br>♂-mHFD: 6.706 ± 0.191<br>♀-CD: 6.638 ± 0.085<br>♀-mHFD: 5.312 ± 0.636	*Diet*Sex*: 6.519<br>*Sex*: 0.6792<br>*Diet*: 0.4359	*Diet*Sex*: **0.0206***<br>*Sex*: 0.4213<br>*Diet*: 0.5180
		NND (mm)	♂-CD: 25.34 ± 0.88<br>♂-mHFD: 23.14 ± 0.37<br>♀-CD: 23.48 ± 0.32<br>♀-mHFD: 26.67 ± 1.66	*Diet*Sex*: 7.725<br>*Sex*: 0.7392<br>*Diet*: 0.2669	*Diet*Sex*: **0.0128***<br>*Sex*: 0.4019<br>*Diet*: 0.6121
		Spacing index (a.u.)	♂-CD: 0.372 ± 0.009<br>♂-mHFD: 0.358 ± 0.005<br>♀-CD: 0.363 ± 0.006<br>♀-mHFD: 0.360 ± 0.008	*Diet*Sex*: 0.5688<br>*Sex*: 0.1806<br>*Diet*: 1.399	*Diet*Sex*: 0.4610<br>*Sex*: 0.6762<br>*Diet*: 0.2532

Supplementary Table 2. Summary of Mixed-effect models test to assess *Diet* and *Sex* main effects as well as *Diet*Sex* interaction effect of ultrastructure analysis of capillaries. Data are presented as Mean ± SEM for each treatment group. *F* and *P* values are given for main and interactions effects (n=93-95 capillaries/diet/sex). ♂: male, ♀: female, a.u.: arbitrary unit, CD: control diet, mHFD: maternal high-fat diet, SEM: standard error of the mean.

**Parameters**		**Mean ± SEM**	***F***	***P***
Endothelium	Area (μm^2)	♂-CD: 5.784±0.359<br>♂-mHFD: 5.830±0.332<br>♀-CD: 5.656±0.376<br>♀-mHFD: 5.295±0.293	*Diet*Sex*: 0.3538<br>*Sex*: 0.9429<br>*Diet*: 0.2114	*Diet*Sex*: 0.5523<br>*Sex*: 0.3322<br>*Diet:* 0.6459
	Basal membrane thickness (nm)	♂-CD: 82.57±2.32<br>♂-mHFD: 75.89±2.25<br>♀-CD: 76.24±2.13<br>♀-mHFD: 76.04±2.12	*Diet*Sex*: 2.156<br>*Sex*: 1.961<br>*Diet*: 2.426	*Diet*Sex*: 0.1428<br>*Sex*: 0.1623<br>*Diet*: 0.1202
	# mitochondria per endothelium volume (/μm^2)	♂-CD: 0.665±0.043<br>♂-mHFD: 0.674±0.053<br>♀-CD: 0.658±0.056	*Diet*Sex*: 0.03531<br>*Sex*: 0.0008057<br>*Diet:* 0.1399	*Diet*Sex*: 0.8511<br>*Sex*: 0.9774<br>*Diet*: 0.7086

		♀-mHFD: 0.685±0.042		
	Ratio of mitochondrial fission/fusion (a.u.)	♂-CD: 0.034±0.010<br>♂-mHFD: 0.023±0.017<br>♀-CD: 0.028±0.010<br>♀-mHFD: 0.017±0.011	*Diet***Sex*: 2.719e-6<br>*Sex*: 0.2000<br>*Diet*: 0.8210	*Diet***Sex*: 0.9987<br>*Sex*: 0.6550<br>*Diet*: 0.3655
	# tight junctions per endothelium volume (/µm²)	♂-CD: 0.092±0.007<br>♂-mHFD: 0.082±0.007<br>♀-CD: 0.076±0.006<br>♀-mHFD: 0.063±0.006	*Diet***Sex*: 0.04699<br>*Sex*: 6.954<br>*Diet*: 3.196	*Diet***Sex*: 0.8285<br>***Sex*: 0.0087****<br>*Diet*: 0.0746
	Average length of tight junctions (nm)	♂-CD:736.3±49.9<br>♂-mHFD: 793.2±61.9<br>♀-CD: 782.8±57.6<br>♀-mHFD: 773.4±63.6	*Diet***Sex*: *0.3193*<br>*Sex*: 0.05137<br>*Diet*: 0.1637	*Diet***Sex*: 0.5727<br>*Sex*: 0.8209<br>*Diet*: 0.6862
	Average thickness of tight junctions (nm)	♂-CD: 176.7±7.1<br>♂-mHFD: 181.5±8.0<br>♀-CD: 177.2±8.0<br>♀-mHFD: 174.9±7.7	*Diet***Sex*: 0.2116<br>*Sex*: 0.1524<br>*Diet*: 0.02316	*Diet***Sex*: 0.6461<br>*Sex*: 0.6967<br>*Diet*: 0.8792
Pericyte	# pericytic process per basal membrane length (/µm)	♂-CD: 0.152±0.011<br>♂-mHFD: 0.155±0.008<br>♀-CD: 0.141±0.010<br>♀-mHFD: 0.132±0.007	*Diet***Sex*: 0.4568<br>*Sex*: 3.550<br>*Diet*: 0.1232	*Diet***Sex*: 0.4996<br>*Sex*: 0.0603<br>*Diet*: 0.7258
	# of lipidic inclusion per pericytic process	♂-CD: 0.011±0.011<br>♂-mHFD: 0.000±0.000<br>♀-CD: 0.011±0.011<br>♀-mHFD: 0.032±0.024	*Diet***Sex*: 1.324<br>*Sex*: 1.306<br>*Diet*: 0.1555	*Diet***Sex*: 0.2507<br>*Sex*: 0.2539<br>*Diet*: 0.6936
Other perivascular cell	# of perivascular cellular process per endothelium length (/µm)	♂-CD: 0.007±0.003<br>♂-mHFD: 0.008±0.003<br>♀-CD: 0.004±0.002<br>♀-mHFD: 0.013±0.005	*Diet***Sex*: 1.395<br>*Sex*: 0.06352<br>*Diet*: 2.722	*Diet***Sex*: 0.2384<br>*Sex*: 0.8012<br>*Diet*: 0.0998
	# of lipidic inclusion per perivascular cellular process	♂-CD: 0.043±0.043<br>♂-mHFD: 0.140±0.140<br>♀-CD: 0.000±0.000	*Diet***Sex*: 0.1326<br>*Sex*: 0.8752<br>*Diet*: 0.8868	*Diet***Sex*: 0.7160<br>*Sex*: 0.3501<br>*Diet*: 0.3470

		♀-mHFD: 0.043±0.034		
Astrocyte	# astrocytic endfeet per endothelium length (/µm)	♂-CD: 0.314±0.014<br>♂-mHFD: 0.293±0.016<br>♀-CD: 0.309±0.017<br>♀-mHFD: 0.292±0.015	*Diet*Sex*: 0.02136<br>*Sex*: 0.04319<br>*Diet*: 1.446	*Diet*Sex*: 0.8839<br>*Sex*: 0.8355<br>*Diet*: 0.2300
	Average area of astrocytic endfeet (µm²)	♂-CD: 2.324±0.263<br>♂-mHFD: 1.569±0.155<br>♀-CD: 1.971±0.209<br>♀-mHFD: 2.261±0.287	*Diet*Sex*: 4.992<br>*Sex*: 0.5213<br>*Diet*: 0.9882	***Diet*Sex*: 0.0261***<br>*Sex*: 0.4707<br>*Diet*: 0.3208
	Ratio of astrocytic endfeet containing glycogen granules (a.u.)	♂-CD: 0.075±0.028<br>♂-mHFD: 0.247±0.045<br>♀-CD: 0.179±0.040<br>♀-mHFD: 0.151±0.037	*Diet*Sex*: 7.001<br>*Sex*: 0.08306<br>*Diet:* 3.594	***Diet*Sex*: 0.0085****<br>*Sex*: 0.9274<br>*Diet*: 0.0588
Microglia	Ratio of microglia in direct contact with the basal membrane	♂-CD: 0.167±0.041<br>♂-mHFD: 0.075±0.030<br>♀-CD: 0.143±0.040<br>♀-mHFD: 0.086±0.031	*Diet*Sex*: 0.2402<br>*Sex*: 0.02970<br>*Diet*: 4.245	*Diet*Sex*: 0.6244<br>*Sex*: 0.8633<br>***Diet*: 0.0402***

Supplementary Table 3. Summary of Mixed-effect models test to assess *Diet* and *Sex* main effects as well as *Diet*Sex* interaction effect of ultrastructure analysis of microglia. Data are presented as Mean ± SEM for each treatment group. *F* and *P* values are given for main and interactions effects (n=77-84 microglia/diet/sex). ♂: male, ♀: female, a.u.: arbitrary unit, CD: control diet, mHFD: maternal high-fat diet, SEM: standard error of the mean.

Parameters		Mean ± SEM	*F*	*P*
Phagolysosomal [illegible]	# primary lysosomes per microglia	♂-CD: 0.893±0.171<br>♂-mHFD: 1.275±0.176<br>♀-CD: 1.026±0.200<br>♀-mHFD: 0.938±0.199	*Diet*Sex*: 1.585<br>*Sex*: 0.2976<br>*Diet*: 0.6224	*Diet*Sex*: 0.2090<br>*Sex*: 0.5858<br>*Diet:* 0.4308

	# secondary lysosomes per microglia	♂-CD: 0.143±0.042<br>♂-mHFD: 0.163±0.045<br>♀-CD: 0.156±0.049<br>♀-mHFD: 0.358±0.088	Diet*Sex: 2.369<br>Sex: 3.091<br>Diet: 3.499	Diet*Sex: 0.1247<br>Sex: 0.0797<br>Diet: 0.0623
	# tertiary lysososmes per microglia	♂-CD: 0.190±0.071<br>♂-mHFD: 0.138±0.050<br>♀-CD: 0.039±0.022<br>♀-mHFD: 0.062±0.027	Diet*Sex: 0.6323<br>Sex: 5.693<br>Diet: 0.1006	Diet*Sex: 0.1153<br>**Sex: 0.0176***<br>Diet: 0.7514
	# lipofuscin granules per microglia	♂-CD: 0.107±0.034<br>♂-mHFD: 0.088±0.032<br>♀-CD: 0.065±0.028<br>♀-mHFD: 0.062±0.027	Diet*Sex: 0.07260<br>Sex: 1.242<br>Diet: 0.1403	Diet*Sex: 0.7878<br>Sex: 0.2659<br>Diet: 0.7082
	# empty endosomes per microglia	♂-CD: 0.095±0.036<br>♂-mHFD: 0.025±0.018<br>♀-CD: 0.130±0.043<br>♀-mHFD: 0.185±0.050	Diet*Sex: 2.644<br>Sex: 6.367<br>Diet: 0.03736	Diet*Sex: 0.1049<br>**Sex: 0.0121***<br>Diet: 0.8469
	# endosomes with digested content per microglia	♂-CD: 0.298±0.067<br>♂-mHFD: 0.350±0.102<br>♀-CD: 0.377±0.072<br>♀-mHFD: 0.531±0.108	Diet*Sex: 0.3252<br>Sex: 2.116<br>Diet: 1.338	Diet*Sex: 0.5689<br>Sex: 0.1467<br>Diet: 0.2483
Metabolism	# mitochondria per microglia	♂-CD: 2.107±0.186<br>♂-mHFD: 3.488±0.351<br>♀-CD: 2.675±0.273<br>♀-mHFD: 2.901±0.293	Diet*Sex: 4.239<br>Sex: 0.001040<br>Diet: 8.206	**Diet*Sex: 0.0403***<br>Sex: 0.9743<br>**Diet: 0.0045****
	# lipidic bodies per microglia	♂-CD: 0.167±0.050<br>♂-mHFD: 0.050±0.025<br>♀-CD: 0.013±0.013<br>♀-mHFD: 0.037±0.021	Diet*Sex: 4.996<br>Sex: 7.006<br>Diet: 2.164	**Diet*Sex: 0.0261***<br>**Sex: 0.0085****<br>Diet: 0.1423
Cellular stress	# mitochondrial anomalies per microglia	♂-CD: 0.440±0.075<br>♂-mHFD: 0.600±0.113	Diet*Sex: 0.02332<br>Sex: 2.632	Diet*Sex: 0.8787<br>Sex: 0.1057

		♀-CD: 0.312±0.079<br>♀-mHFD: 0.444±0.079	*Diet*: 2.781	*Diet:* 0.0964
	Ratio with dilated cisternae of endoplasmic reticulum and Golgi apparatus (a.u.)	♂-CD: 0.929±0.028<br>♂-mHFD: 0.888±0.036<br>♀-CD: 0.792±0.047<br>♀-mHFD: 0.938±0.027	*Diet*Sex*: 7.240<br>*Sex*: 1.515<br>*Diet*: 2.279	**Diet*Sex: 0.0075****<br>*Sex*: 0.2194<br>*Diet:* 0.1321
Interaction with their microenvironment	# contacts with presynaptic terminal	♂-CD: 4.774±0.327<br>♂-mHFD: 6.925±0.375<br>♀-CD: 5.156±0.336<br>♀-mHFD: 4.679±0.320	*Diet*Sex*: 14.92<br>*Sex*: 7.504<br>*Diet*: 6.055	**Diet*Sex: 0.0001*****<br>**Sex: 0.0065****<br>**Diet: 0.0144***
	# contacts with postsynaptic dendrites	♂-CD: 2.583±0.220<br>♂-mHFD: 3.388±0.256<br>♀-CD: 2.857±0.240<br>♀-mHFD: 2.432±0.206	*Diet*Sex*: 7.068<br>*Sex*: 2.173<br>*Diet*: 0.6724	**Diet*Sex: 0.0082****<br>*Sex*: 0.1414<br>*Diet:* 0.4128
	# contacts with myelinated axons	♂-CD: 0.333±0.068<br>♂-mHFD: 0.225±0.067<br>♀-CD: 0.260±0.068<br>♀-mHFD: 0.185±0.050	*Diet*Sex*: 0.07038<br>*Sex*: 0.7934<br>*Diet*: 2.063	*Diet*Sex*: 0.7910<br>*Sex*: 0.3738<br>*Diet:* 0.1519
	# contacts with degraded myelin	♂-CD: 0.145±0.042<br>♂-mHFD: 0.075±0.035<br>♀-CD: 0.117±0.049<br>♀-mHFD: 0.185±0.050	*Diet*Sex*: 2.417<br>*Sex*: 0.8651<br>*Diet*: 0.0002071	*Diet*Sex*: 0.1210<br>*Sex*: 0.3530<br>*Diet:* 0.9885
	Ratio touching a neuronal cell (a.u.)	♂-CD: 0.321±0.051<br>♂-mHFD: 0.325±0.053<br>♀-CD: 0.312±0.059<br>♀-mHFD: 0.519±0.056	*Diet*Sex*: *3.448*<br>*Sex*: 2.819<br>*Diet*: 3.694	*Diet*Sex*: 0.0643<br>*Sex*: 0.0942<br>*Diet:* 0.0555
	Ratio touching an astrocytic cell (a.u.)	♂-CD: 0.024±0.017<br>♂-mHFD: 0.025±0.018<br>♀-CD: 0.039±0.022<br>♀-mHFD: 0.000±0.000	*Diet*Sex*: 1.519<br>*Sex*: 0.09139<br>*Diet*: 1.344	*Diet*Sex*: 0.2187<br>*Sex*: 0.7676<br>*Diet:* 0.2472

	Ratio touching an oligodendrocytic cell (a.u.)	♂-CD: 0.012±0.012<br>♂-mHFD: 0.000±0.000<br>♀-CD: 0.026±0.018<br>♀-mHFD: 0.012±0.012	*Diet*Sex*: 0.004841<br>*Sex*: 1.137<br>*Diet*: 1.062	*Diet*Sex*: 0.9446<br>*Sex*: 0.2871<br>*Diet:* 0.3035
	# microglia-associated extracellular space pockets	♂-CD: 5.298±0.340<br>♂-mHFD: 3.213±0.270<br>♀-CD: 5.221±0.365<br>♀-mHFD: 4.926±0.287	*Diet*Sex*: 7.931<br>*Sex*: 6.628<br>*Diet*: 14.02	***Diet*Sex*: 0.0052****<br>***Sex*: 0.0105***<br>***Diet:* 0.0002*****
	# exophagy events per microglia	♂-CD: 0.369±0.067<br>♂-mHFD: 0.225±0.050<br>♀-CD: 0.182±0.048<br>♀-mHFD: 0.420±0.080	*Diet*Sex*: 9.084<br>*Sex*: 0.003524<br>*Diet*: 0.5488	***Diet*Sex*: 0.0028****<br>*Sex*: 0.9527<br>*Diet:* 0.4594

Supplementary Table 4. Summary of ANOVAs test to assess *Diet* and *Sex* main effects as well as *Diet*Sex* interaction effect of behavioural data. Data are presented as Mean ± SEM for each treatment group. *F* and *P* values are given for main and interactions effects (n=6-10 litters/diet/sex, N=8-12 animals/group). ♂: male, ♀: female, a.u.: arbitrary unit, CD: control diet, mHFD: maternal high-fat diet, SEM: standard error of the mean.

**Parameters**		**Mean ± SEM**	***F***	***P***
Marbles burying	Number of buried marbles	♂-CD: 5.055 ± 1.320<br>♂-mHFD: 9.111 ± 0.553<br>♀-CD: 4.291 ± 1.493<br>♀-mHFD: 7.134 ± 0.939	*Diet*Sex*: 0.3047<br>*Sex*: 1.555<br>*Diet*: 9.852	*Diet*Sex*: 0.5852<br>*Sex*: 0.2224<br>*Diet*: **0.0039****
Open field	Distance travelled (cm)	♂-CD: 995.6 ± 66.9<br>♂-mHFD: 1167.2 ± 135.9<br>♀-CD: 1403.6 ± 139.0<br>♀-mHFD: 1363.3 ± 125.8	*Diet*Sex*: 0.7673<br>*Sex*: 6.241<br>*Diet*: 0.2946	*Diet*Sex*: 0.3866<br>*Sex*: **0.0169***<br>*Diet*: 0.5905
	Moving time (min)	♂-CD: 1.795 ± 0.105<br>♂-mHFD: 2.216 ± 0.242<br>♀-CD: 2.556 ± 0.212<br>♀-mHFD: 2.436 ± 0.226	*Diet*Sex*: 1.688<br>*Sex*: 5.545<br>*Diet*: 0.5216	*Diet*Sex*: 0.2016<br>*Sex*: **0.0238***<br>*Diet*: 0.4746
	Center time (min)	♂-CD: 1.946 ± 0.311	*Diet*Sex:* 3.793	*Diet*Sex*: 0.0589

		♂-mHFD: 2.865 ± 0.349<br>♀-CD: 2.781 ± 0.438<br>♀-mHFD: 2.325 ± 0.323	Sex: 0.1745<br>Diet: 0.4304	Sex: 0.6785<br>Diet: 0.5157
	Number of revolutions	♂-CD: 9.375 ± 1.194<br>♂-mHFD: 6.545 ± 0.755<br>♀-CD: 9.417 ± 1.151<br>♀-mHFD: 8.818 ± 1.577	Diet*Sex: 0.8201<br>Sex: 0.8825<br>Diet: 1.936	Diet*Sex: 0.3709<br>Sex: 0.3534<br>Diet: 0.1722
	Number of stereotypies	♂-CD: 10.47 ± 0.25<br>♂-mHFD: 10.68 ± 0.23<br>♀-CD: 10.88 ± 0.24<br>♀-mHFD: 10.92 ± 0.15	Diet*Sex: 0.1445<br>Sex: 2.093<br>Diet: 0.3242	Diet*Sex: 0.7059<br>Sex: 0.1562<br>Diet: 0.5724
	Number of stereotypies count	♂-CD: 178.1 ± 17.1<br>♂-mHFD: 192.8 ± 17.1<br>♀-CD: 218.1 ± 13.4<br>♀-mHFD: 192.6 ± 14.7	Diet*Sex: 1.521<br>Sex: 1.488<br>Diet: 0.1092	Diet*Sex: 0.2250<br>Sex: 0.2300<br>Diet: 0.7429
	Duration of stereotypies	♂-CD: 3.067 ± 0.258<br>♂-mHFD: 3.112 ± 0.228<br>♀-CD: 3.265 ± 0.214<br>♀-mHFD: 2.922 ± 0.155	Diet*Sex: 0.7801<br>Sex: 0.0002930<br>Diet: 0.4599	Diet*Sex: 0.3827<br>Sex: 0.9864<br>Diet: 0.5018
Elevated plus-maze	Time spent in closed arms (sec)	♂-CD: 206.2 ± 11.8<br>♂-mHFD: 196.8 ± 9.9<br>♀-CD: 206.4 ± 5.3<br>♀-mHFD: 203.1 ± 7.9	Diet*Sex: 0.1104<br>Sex: 0.1220<br>Diet: 0.4802	Diet*Sex: 0.7421<br>Sex: 0.7294<br>Diet: 0.4938
	Time spent in open arms (sec)	♂-CD: 32.83 ± 7.73<br>♂-mHFD: 61.01 ± 24.42<br>♀-CD: 40.12 ± 6.47<br>♀-mHFD: 48.43 ± 17.16	Diet*Sex: 0.3683<br>Sex: 0.02612<br>Diet: 1.240	Diet*Sex: 0.5482<br>Sex: 0.8726<br>Diet: 0.2737
	Anxiety index (a.u.)	♂-CD: 0.113 ± 0.038<br>♂-mHFD: 0.143 ± 0.033<br>♀-CD: 0.135 ± 0.030<br>♀-mHFD: 0.166 ± 0.026	Diet*Sex: 0.0001405<br>Sex:0.4857<br>Diet: 0.8940	Diet*Sex: 0.9906<br>Sex: 0.4914<br>Diet: 0.3522
Three-chambers test	Social preference (a.u.)	♂-CD: 1.150 ± 0.127<br>♂-mHFD: 1.256 ± 0.166<br>♀-CD: 1.270 ± 0.187<br>♀-mHFD: 1.159 ± 0.126	Diet*Sex: 0.4808<br>Sex: 0.005218<br>Diet: 0.0002941	Diet*Sex: 0.4942<br>Sex: 0.9430<br>Diet: 0.9864
	Social novelty preference (a.u.)	♂-CD: 1.104 ± 0.160<br>♂-mHFD: 1.096 ± 0.253<br>♀-CD: 1.142 ± 0.260<br>♀-mHFD: 1.196 ± 0.198	Diet*Sex: 0.01696<br>Sex: 0.08394<br>Diet: 0.009322	Diet*Sex: 0.8974<br>Sex: 0.7744<br>Diet: 0.9239
Novel object recogn	Acquisition: exploration time (sec)	♂-CD: 53.81 ± 4.49<br>♂-mHFD: 50.29 ± 4.53	Diet*Sex: 0.0006174<br>Sex: 0.2768	Diet*Sex: 0.9804<br>Sex: 0.6029

		♀-CD: 55.81 ± 5.05<br>♀-mHFD: 52. 49 ± 2.41	*Diet*: 0.7361	*Diet*: 0.3982
	Recognition: exploration time (sec)	♂-CD: 62.79 ± 3.46<br>♂-mHFD: 61.88 ± 2.19<br>♀-CD: 68.13 ± 5.40<br>♀-mHFD: 66.85 ± 1.87	*Diet*Sex*: 0.003415<br>*Sex*: 2.438<br>*Diet*: 0.1097	*Diet*Sex*: 0.9538<br>*Sex*: 0.1301<br>*Diet*: 0.7430
Prepulse inhibition	PP3: % of inhibition	♂-CD: 27.90 ± 6.10<br>♂-mHFD: 24.88 ± 3.84<br>♀-CD: 30.96 ± 3.56<br>♀-mHFD: 22.34 ± 5.85	*Diet*Sex*: 0.3772<br>*Sex*: 2.825<br>*Diet*: 0.1567	*Diet*Sex*: 0.5434<br>*Sex*: 0.1026<br>*Diet*: 0.6948
	PP6: % of inhibition	♂-CD: 40.42 ± 5.29<br>♂-mHFD: 44.52 ± 6.46<br>♀-CD: 44.56 ± 5.19<br>♀-mHFD: 49.12 ± 3.71	*Diet*Sex*: 0.08449<br>*Sex*: 0.1398<br>*Diet*: 0.1351	*Diet*Sex*: 0.7732<br>*Sex*: 0.7110<br>*Diet*: 0.7157
	PP9: % of inhibition	♂-CD: 52.43 ± 5.23<br>♂-mHFD: 50.51 ± 6.64<br>♀-CD: 49.86 ± 5.85<br>♀-mHFD: 48.69 ± 6.45	*Diet*Sex*: 0.1058<br>*Sex*: 0.1709<br>*Diet*: 0.09708	*Diet*Sex*: 0.7471<br>*Sex*: 0.6820<br>*Diet*: 0.7574
	PP12: % of inhibition	♂-CD: 44.11 ± 7.26<br>♂-mHFD: 62.64 ± 4.29<br>♀-CD: 56.82 ± 5.88<br>♀-mHFD: 58.07 ± 4.63	*Diet*Sex*: 1.300<br>*Sex*: 0.3694<br>*Diet*: 2.414	*Diet*Sex*: 0.2627<br>*Sex*: 0.5476<br>*Diet*: 0.1301
	PP15: % of inhibition	♂-CD: 59.34 ± 5.48<br>♂-mHFD: 56.30 ± 6.84<br>♀-CD: 63.63 ± 4.33<br>♀-mHFD: 68.30 ± 3.85	*Diet*Sex*: 1.413<br>*Sex*: 2.102<br>*Diet*: 0.008190	*Diet*Sex*: 0.2433<br>*Sex*: 0.1568<br>*Diet*: 0.9285
	Mean: % of inhibition	♂-CD: 49.08 ± 5.24<br>♂-mHFD: 53.49 ± 5.41<br>♀-CD: 53.72 ± 4.11<br>♀-mHFD: 54.69 ± 4.04	*Diet*Sex*: 0.1292<br>*Sex*: 0.3696<br>*Diet*: 0.3148	*Diet*Sex*: 0.7214<br>*Sex*: 0.5471<br>*Diet*: 0.5783
	Amplitude of startle response (a.u.)	♂-CD: 48.41 ± 6.19<br>♂-mHFD: 66.70 ± 12.65<br>♀-CD: 55.59 ± 7.77<br>♀-mHFD: 67.44 ± 10.54	*Diet*Sex*: 0.1168<br>*Sex*: 0.1766<br>*Diet*: 2.558	*Diet*Sex*: 0.7346<br>*Sex*: 0.6769<br>*Diet*: 0.1187

Supplementary Table 5. Detailed diet composition of control chow vs high-fat diet. The different components of the diet are presented as either a percentage of food or a quantity (g, mg or µg) per kilogram of food. CD: control diet, HFD: high-fat diet, N/A: not available. [1] Mineral mix AIN-93G-MX (cat# 94046, Teklad), [2] Vitamins mix AIN-93-VX (cat# 94047, Teklad).

	units	CD	HFD
**Macronutrients**			
Crude protein	%	14.3	23.4
Fats	%	4	34.3
Lard	g/kg	N/A	310
Soybean Oil	g/kg	N/A	30
Cellulose	g/kg	N/A	65.5
Carbohydrate	%	48	27.3
Crude fiber	%	4.1	N/A
Neutral detergent fiber	%	18	N/A
Ash	%	4.7	N/A
**Mineral**			**48**[1]
Calcium	%	0.7	N/A
Phosphorus	%	0.6	N/A
Sodium	%	0.1	N/A
Potassium	%	0.6	N/A
Chloride	%	0.3	N/A
Magnesium	%	0.2	N/A
Zinc	mg/kg	70	N/A
Manganese	mg/kg	100	N/A
Cooper	mg/kg	15	N/A
Iodine	mg/kg	6	N/A
Iron	mg/kg	175	N/A
Selenium	mg/kg	0.23	N/A
Calcium phosphate, dibasic	g/kg	-	3.4
**Amino acids**			
Aspartic acid	%	0.9	N/A
Glutamic acid	%	2.9	N/A
Alanine	%	0.9	N/A
Glycine	%	0.7	N/A
Threonine	%	0.5	N/A
Proline	%	1.2	N/A
Serine	%	0.7	N/A
Leucine	%	1.4	N/A
Isoleucine	%	0.6	N/A
Valine	%	0.7	N/A
Phenylalanine	%	0.7	N/A
Tyrosine	%	0.4	N/A
Methionine	%	0.3	N/A
Cystine	%	0.3	0.04
Lysine	%	0.7	N/A
Histidine	%	0.4	N/A
Arginine	%	0.8	N/A
Tryptophan	%	0.2	N/A

**Vitamins**			**21**[2]
Vitamin A	mg/kg	1.8	N/A
Vitamin D3	µg/kg	15	N/A
Vitamin E	mg/kg	80.4	N/A
Vitamin K	mg/kg	20	N/A
Vitamin B1 (thiamin)	mg/kg	12	N/A
Vitamin B2 (riboflavin)	mg/kg	6	N/A
Niacin (nicotinic acid)	mg/kg	54	N/A
Vitamin B6 (pyridoxine)	mg/kg	10	N/A
Pantothenic acid	mg/kg	17	N/A
Vitamin B12 (cyanocobalamin)	mg/kg	0.03	N/A
Biotin	mg/kg	0.26	N/A
Folate	mg/kg	2	N/A
Choline	mg/kg	1030	3000 **choline bitartrate*
**Fatty acids**			
C16:0 Palmitic	%	0.5	N/A
C18:0 Stearic	%	0.1	N/A
C18:1ω9 Oleic	%	0.7	N/A
C18:2ω6 Linoleic	%	2	N/A
C18:3ω3 Linolenic	%	0.1	N/A
Total saturated	%	0.6	36
Total monounsaturated	%	0.7	41
Total polyunsaturated	%	2.1	23
**Other**			
Blue food color	g/kg	-	0.1
Casein	g/kg	-	265
Maltodextrin	g/kg	-	160
Sucrose	g/kg	-	90
