# Supplementary material for: Maternal high-fat diet in mice induces cerebrovascular, microglial and long-term behavioural alterations in offspring
Source: Commun Biol. 2022 Jan 11;5:26. doi: 10.1038/s42003-021-02947-9 (PMC8752761; doi:10.1038/s42003-021-02947-9)
Supplement: Supplementary file 3 — Description of Additional Supplementary Files [file 42003_2021_2947_MOESM3_ESM.pdf]

Description of Additional Supplementary Files

File name: Supplementary Data 1

Description: Summary of ANOVAs test to assess Diet and Sex main effects as well as Diet*Sex interaction effect of 2D and 3D analyses of the neurovascular unit data. Data are presented as Mean ± SEM for each treatment group. F and P values are given for main and interactions effects (n=5-6 animals/diet/sex, 2-3 sections). ♂: male, ♀: female, a.u.: arbitrary unit, CD: control diet, mHFD: maternal high-fat diet, SEM: standard error of the mean.
